# Relative efficacy of minoxidil in combination with other treatments for androgenic alopecia: a network meta-analysis based on randomized controlled trials

**DOI:** 10.3389/fmed.2025.1638496

**Published:** 2025-09-17

**Authors:** Yinfeng Xia, Hong Chen, Yongsong Chen, Zhiyong Chen

**Affiliations:** ^1^Department of Burns, Plastic Surgery and Cosmetology, Chongqing University Fuling Hospital, Chongqing University, Chongqing, China; ^2^Department of Hepatobiliary Surgery, The Second Affiliated Hospital of Chongqing Medical University, Chongqing, China

**Keywords:** androgenic alopecia, minoxidil, minoxidil combination therapy, male pattern hair loss, female pattern hair loss

## Abstract

**Background:**

Androgenetic alopecia is the most prevalent form of progressive hair loss. Minoxidil is widely regarded as a standard treatment for this condition. Consequently, we assessed the effectiveness of minoxidil in combination with other pharmacological agents for the treatment of androgenetic alopecia.

**Methods:**

A comprehensive search was conducted across four databases–PubMed, Embase, Web of Science, and Cochrane Library–on December 10, 2024. Eligible studies were selected based on the PICOS framework. Data extraction and synthesis were carried out using a Bayesian network meta-analysis, focusing on mean difference and sample size data. League tables and Surface Under the Cumulative Ranking (SUCRA) values were employed to evaluate the relative efficacy of the interventions.

**Results:**

Among the 20 study groups analyzed, the combination of platelet-rich plasma and basic fibroblast growth factor with minoxidil demonstrated the highest overall efficacy (SUCRA = 93.06%). This combination resulted in a mean increase in hair density of 35.12 hairs/cm^2^ compared to the group treated with minoxidil alone. In male subgroups, finasteride combined with minoxidil was the most effective treatment (SUCRA = 80.18%). Among seven combination therapies for females, microneedle with minoxidil proved most effective (SUCRA = 87.18%).

**Conclusion:**

This study establishes a clinically actionable hierarchy of minoxidil-based combination therapies, providing evidence-based guidance for dermatologists to optimize androgenetic alopecia management.

**Systematic review registration:**

https://www.crd.york.ac.uk/PROSPERO/view/CRD42024623164, identifier CRD42024623164.

## Introduction

Androgenetic alopecia (AGA), known as male or female pattern hair loss, is the most common form of alopecia worldwide, characterized by progressive hair loss post-puberty and worsening with age. While AGA affects both genders, it is more prevalent in men and impacts quality of life, often leading to low self-esteem (1). The condition is linked to the distribution of androgen receptors in the scalp, differing between males and females, male pattern baldness (MPHL) mainly affects the crown and frontal areas of the scalp, accompanied by a receding hairline, while female pattern baldness (FPHL) is diffuse hair loss, with the hairline often remaining normal (2–4). Despite the development of numerous therapies for androgenetic alopecia (AGA), many fail to meet expectations, and AGA remains a significant concern for affected individuals (5). Currently, the Food and Drug Administration (FDA) has approved two medications for AGA treatment: oral finasteride and topical minoxidil (6). However, the limited efficacy of these treatments when used independently has prompted research into the effectiveness of minoxidil when combined with other therapies. Studies indicate that combination therapies are more effective than monotherapy (7–10). Nonetheless, there is a paucity of comprehensive literature comparing the advantages and disadvantages of various minoxidil combination therapies. Therefore, this article aims to systematically compare and rank different minoxidil combination therapies, providing a valuable reference for the clinical management of androgenetic alopecia.

## Method

The protocol for our work was published in the International Platform of Registered Systematic Review and Meta-Analysis Protocols (PROSPERO) database under the ID: CRD42024623164. Our work also followed the Preferred Reporting Items for Systematic Reviews and Meta-Analyses (PRISMA-NMA) guidelines (11).

### Identification of eligible studies

The study was designed in accordance with the PICOS framework, focusing on patients with androgenic alopecia (P). The interventions compared included minoxidil in combination with other therapies versus minoxidil alone (I/C), with the primary outcome being the change in hair density at 24 weeks (O). All included studies were randomized controlled trials (S). Given the proximity of 24 weeks to 6 months, results from studies with a 6-months follow-up were incorporated into the final analysis. This outcome was selected due to its common usage as an endpoint and its relative objectivity.

A comprehensive search was conducted across four databases–PubMed, Embase, Web of Science, and the Cochrane Library–on December 10, 2024, with a publication cutoff of December 1, 2024. The search strategy employed both subject-specific and free-text terms. Literature selection was performed independently by two authors, who screened titles, abstracts, and full texts. Discrepancies were assessed and resolved by a third author. Data extraction included details such as author, year of publication, patient demographics (sex and age), severity of androgenetic alopecia, and treatment regimen.

### Statistical analyses

In this study, direct comparisons between interventions across trials are illustrated using network plots, which are graphs consisting of nodes and edges. Each node represents a specific intervention, while an edge, depicted as a line connecting two nodes, signifies a direct comparison between the two interventions in a head-to-head trial. The thickness of an edge indicates the number of direct comparisons between the corresponding nodes.

A Bayesian network analysis was conducted, employing 50,000 iterations and a random effects model. All analyses were executed using RStudio software. The Surface Under the Cumulative Ranking (SUCRA) value was calculated for each intervention, allowing for the ranking of interventions and the generation of an optimized SUCRA line chart. A 95% confidence interval was estimated for each comparison measure, with a *p*-value of less than 0.05 denoting statistical significance. The quality of evidence in the study was assessed using the latest version of the Cochrane Collaboration’s Risk of Bias 2 tool.

## Results

**FIGURE 1:**
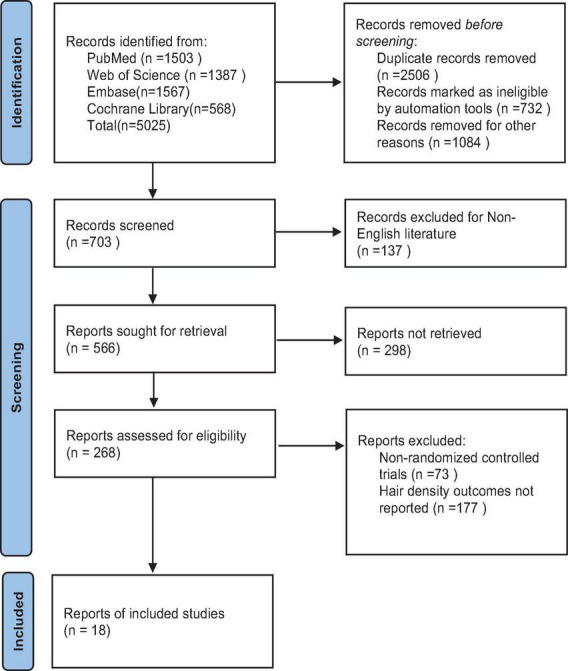
Screening flow chart.

**FIGURE 2:**
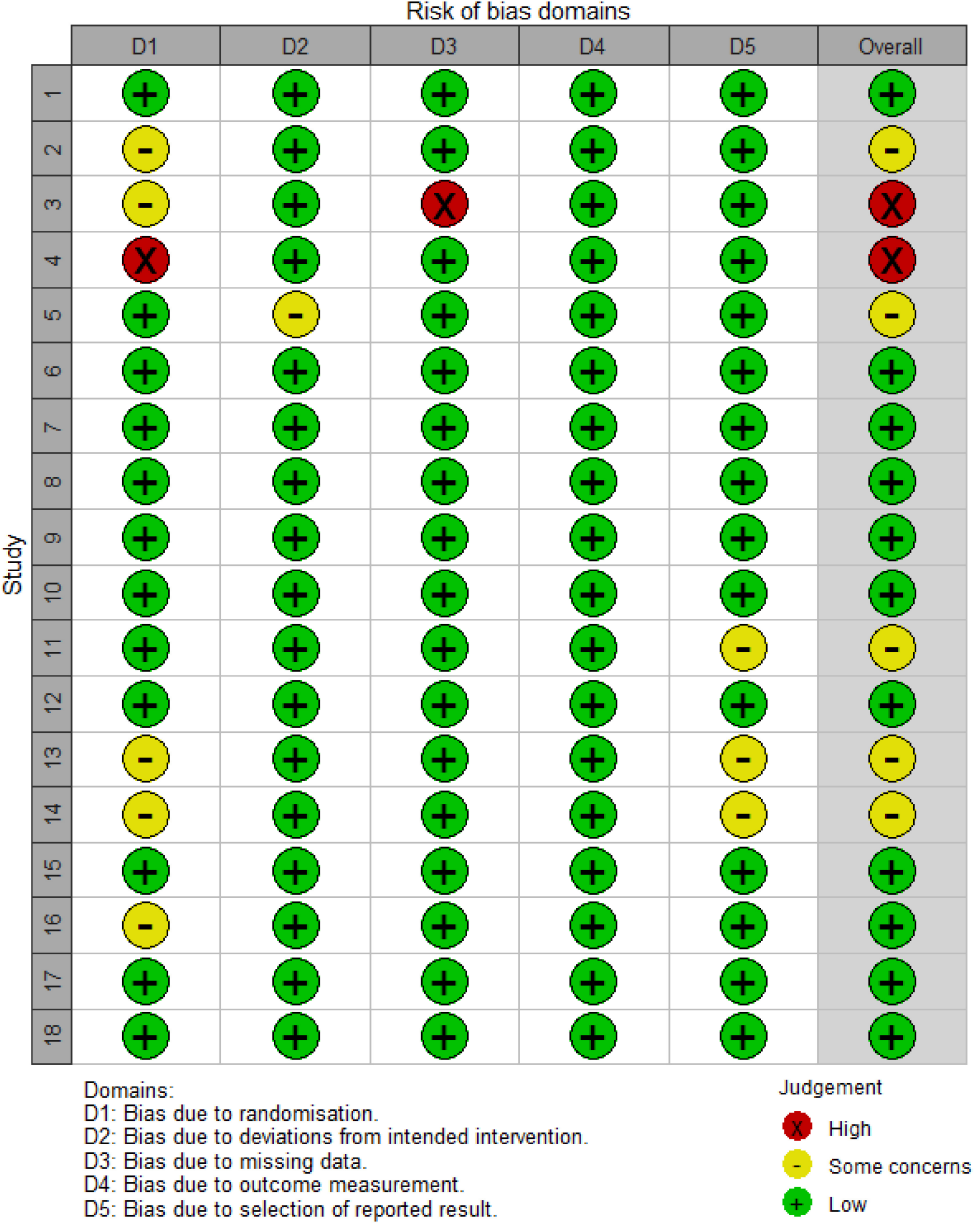
Risk of bias assessment chart. Literature Information: 1. Faghihi et al. (12); 2. Ferrara et al. (13); 3. Yang et al. (14); 4. Khattab and Bessar (16); 5. Singh et al. (17); 6. Wu et al. (19); 7. Suchonwanit et al. (24); 8, 9. Bao et al. (20); 10, 11. Suchonwanit et al. (25); 12. Rossi et al. (26); 13. Faghihi et al. (27); 14. Zhang et al. (22); 15. Bassiouny et al. (28); 16. Alves and Grimalt (18); 17. Tan et al. (29); 18. Liang et al. (23).

**FIGURE 3:**
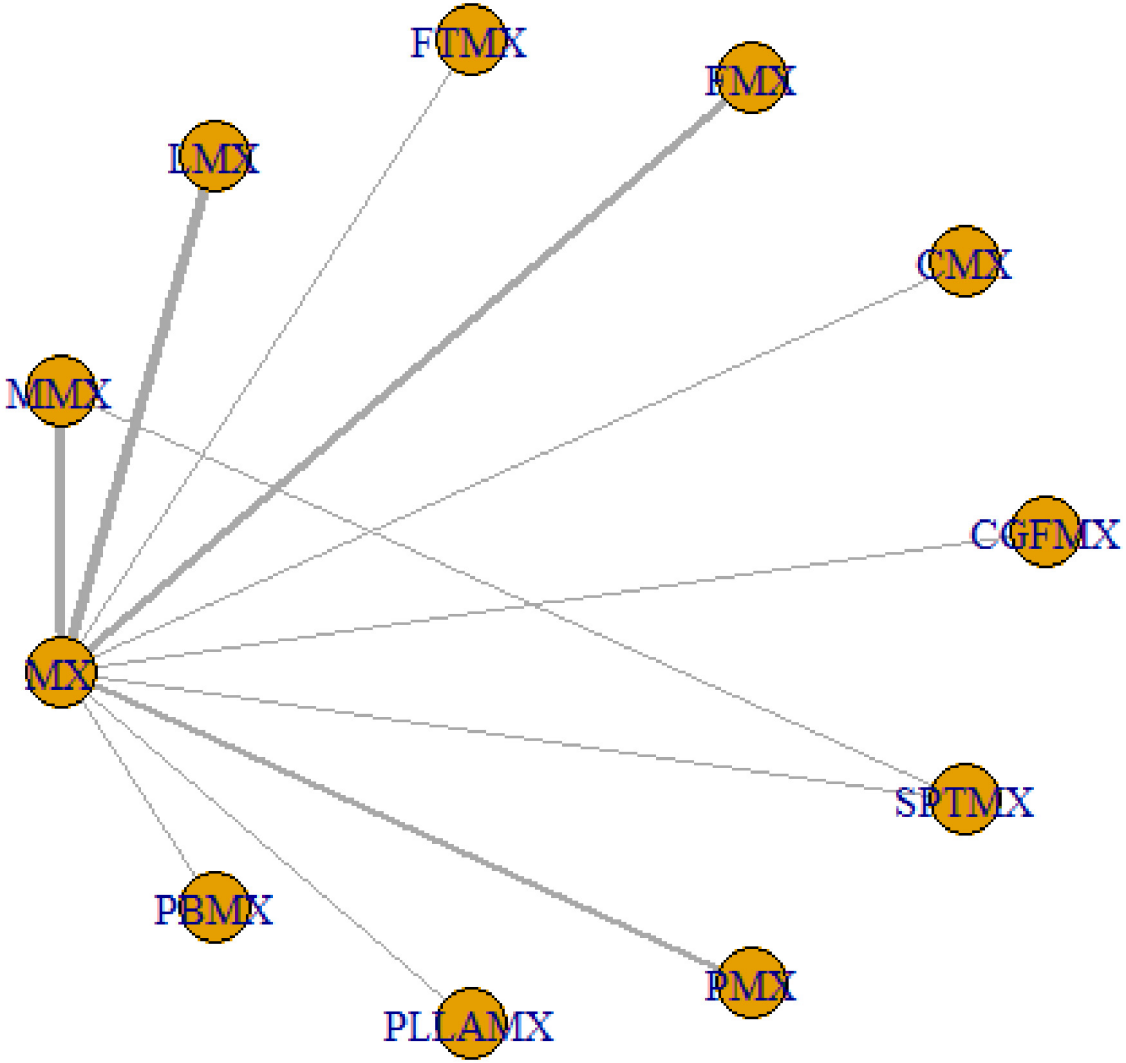
Network plot for mixed groups. One node corresponds to a given intervention; an edge is represented by a line between the two nodes, which corresponds to a direct comparison of the two interventions in a head-on trial. The thickness of an edge corresponds to the number of direct comparisons between the respective nodes.

A total of 5,025 studies were identified, of which 18 were selected for inclusion in the analysis following a rigorous screening process (Figure 1). The results of the risk of bias assessment for each study are presented in Figure 2. The quality of evidence for each intervention is detailed in Supplementary Tables 1–3. The study encompassed 729 patients, 20 intervention comparisons, and 10 combinations involving minoxidil. The interventions included: minoxidil combined with low-level light therapy (LMX) (*n* = 4), monofilament thread therapy with minoxidil (PLLAMX) (*n* = 1), platelet-rich plasma with minoxidil (PMX) (*n* = 2), platelet-rich plasma plus basic fibroblast growth factor combined with minoxidil (PBMX) (*n* = 1), microneedling with minoxidil (MMX) (*n* = 4), finasteride with minoxidil (FMX) (*n* = 3), flutamide with minoxidil (FTMX) (*n* = 1), cetirizine with minoxidil (CMX) (*n* = 1), concentrated growth factors with minoxidil (CGFMX) (*n* = 1), and spironolactone with minoxidil (SPTMX) (*n* = 1). The characteristics of each research group are detailed in Table 1. A network plot illustrating the various interventions is shown in Figure 3. Minoxidil monotherapy was used as the reference group, as each study’s control group consisted of minoxidil alone.

**TABLE 1:** Basic information sheets.

Study	Age	Sample size (percentage of male patients)	Severity mild/moderate/severe	Design (whole-scalp or split-scalp)	Treatment
Faghihi et al. (12)	NA	23	NA	Whole	Topical minoxidil 5%, 20 drops, twice per day for 6 months, low level light therapy (10–50 MW power and a 785 nm wavelength) per week for 24 weeks;
		22	NA	Whole	Topical minoxidil 5%, 20 drops, twice per day for 6 months
Ferrara et al. (13)	41.7 ± 6.76	19 (1)	0/9/10	Half	5 mw of 660 nm light to the irradiated side when used for 24 min per day and 1 mL of 5% topical minoxidil for 6 months
		19 (1)	0/9/10	Half	1 mL of 5% topical minoxidil for 6 months
Yang et al. (14)	29.8 ± 5.74	30 (0)	NA	Whole	1 ml 2% topical minoxidil solution twice daily for 24 weeks, laser hamlet treatment 20 min every other day for 24 weeks
		30 (0)	NA	Whole	1 ml 2% topical minoxidil solution twice daily for 24 weeks
Suchonwanit et al. (15)	35.4 ± 10.3	29 (1)	0/16/13	Half	1 mL of 5% topical minoxidil solution twice daily for 24 weeks and 1550-nm fractional erbium-glass laser treatment at 2-weeks intervals for a total of 12 sessions
		29 (1)	0/16/13	Half	1 mL of 5% topical minoxidil solution twice daily for 24 weeks
Khattab and Bessar (16)	32 (21–49)	27 (0)	NA	Half	1 mL of 2% topical minoxidil solution twice daily for 6 months, ploy-L-lactic acid threads were implemented in the dermal layer.
		27 (0)	NA	Half	1 mL of 2% topical minoxidil solution twice daily for 6 months,
Singh et al. (17)	26.1 ± 4.2	20 (1)	NA	Whole	1 mL of 5% topical minoxidil solution twice daily for 3 months, intradermal injection of PRP monthly for 3 months
		17 (1)	NA	Whole	1 mL of 5% topical minoxidil solution twice daily for 3 months
Alves and Grimalt (18)	39.9 (18–65)	13 (0)	3/9/1	Half	1 mL 5% topical minoxidil solution twice daily and intradermal injection of PRP monthly for 6 months
		13 (0)	3/9/1	Half	1 mL 5% topical minoxidil solution twice daily for 6 months
Wu et al. (19)	26.1 ± 4.2	20 (0.48)	NA	Whole	1 mL of 5% topical minoxidil solution twice daily for 6 months, three PRP, BFGF treatment sessions at 1-month intervals
		17 (0.64)	NA	Whole	1 mL of 5% topical minoxidil solution twice daily for 6 months,
Bao et al. (20)	35.2 ± 3.3	20 (1)	0/11/9	Whole	1 mL of 5% topical minoxidil solution twice daily for 24 weeks and microneedle 2-weeks intervals for a total of 12 times
	34.7 ± 6.9	18 (1)	0/11/7	Whole	1 mL of 5% topical minoxidil solution twice daily for 24 weeks
Bao et al. (21)	36.33 ± 8.04	25 (1)	0/13/12	Whole	1 mL of 5% topical minoxidil solution twice daily for 24 weeks and microneedle 2-weeks intervals for a total of 12 times
	37.01 ± 8.43	23 (1)	0/13/10	Whole	1 mL of 5% topical minoxidil solution twice daily for 24 weeks
Zhang et al. (22)	31.68 ± 4.93	20 (0)	NA	Whole	1 ml of topical 2% minoxidil solution twice a day and 24 sessions of weekly microneedle over a total period of 24 weeks
	30.05 ± 5.46	20 (0)	NA	Whole	1 mL of 2% topical minoxidil solution twice daily for 24 weeks
Liang et al. (23)	30.83 ± 6.28	40 (0)	NA	Whole	Microneedle treatments with the delivery of 5% topical minoxidil every 2 weeks and 1 ml of topical 5% topical minoxidil once daily for 24 weeks
	31.62 ± 6.29	37 (0)	NA	Whole	Oral SPT of 80–100 mg/day and 1 ml of topical 5% minoxidil once daily for 24 weeks
	31.08 ± 6.87	40 (0)	NA	Whole	1 ml of topical 5% minoxidil once daily for 24 weeks
Suchonwanit et al. (24)	56.8 ± 6.6	15 (0)	5/8/2	Whole	1 mL of 3% topical minoxidil and 0.25% finasteride solution twice daily for 24 weeks
	59.8 ± 7.7	15 (0)	4/8/3	Whole	1 ml of topical 3% minoxidil once daily for 24 weeks
Suchonwanit et al. (25)	39.3 ± 11.9	19 (1)	0/12/7	Whole	1 mL of 3% topical minoxidil and 0.25% finasteride solution twice daily for 24 weeks
	44.4 ± 12.5	18 (1)	014/4	Whole	1 ml of topical 3% minoxidil once daily for 24 weeks
Rossi and Caro (26)	25.3 ± 2.6	19 (1)	11/5/3	Whole	5% topical minoxidil in the morning and 0.25% topical finasteride spray in the evening
	23.5 ± 2.2	11 (1)	10/1/0	Whole	5% topical minoxidil twice daily
Faghihi et al. (27)	27.15 ± 5.29	20 (0.3)	NA	Whole	1 mL of 5% topical minoxidil and 2% flutamide solution twice daily for 6 months
	27.05 ± 4.75	20 (0.55)	NA	Whole	1 ml of topical 5% minoxidil twice daily for 24 weeks
Bassiouny et al. (28)	38.61 ± 8.74	26 (0)	NA	Whole	1 ml of topical minoxidil (5%) once daily in the morning and 1 ml of topical cetirizine (1%) once daily in the evening for 24 weeks
	36.74 ± 9.84	27 (0)	NA	Whole	1 ml of topical minoxidil (5%) once daily in the morning and 1 ml of placebo once daily in the evening
Tan et al. (29)	NA	16 (1)	NA	Half	1 mL 5% topical minoxidil solution twice daily for 24 weeks and intradermal injection of CGF (2–3 ml) at 0, 4, 8 weeks
		16 (1)	NA	Half	1 mL 5% topical minoxidil solution twice daily for 24 weeks and intradermal injection of placebo normal saline (2–3 ml) at 0, 4, 8 weeks

**FIGURE 4:**
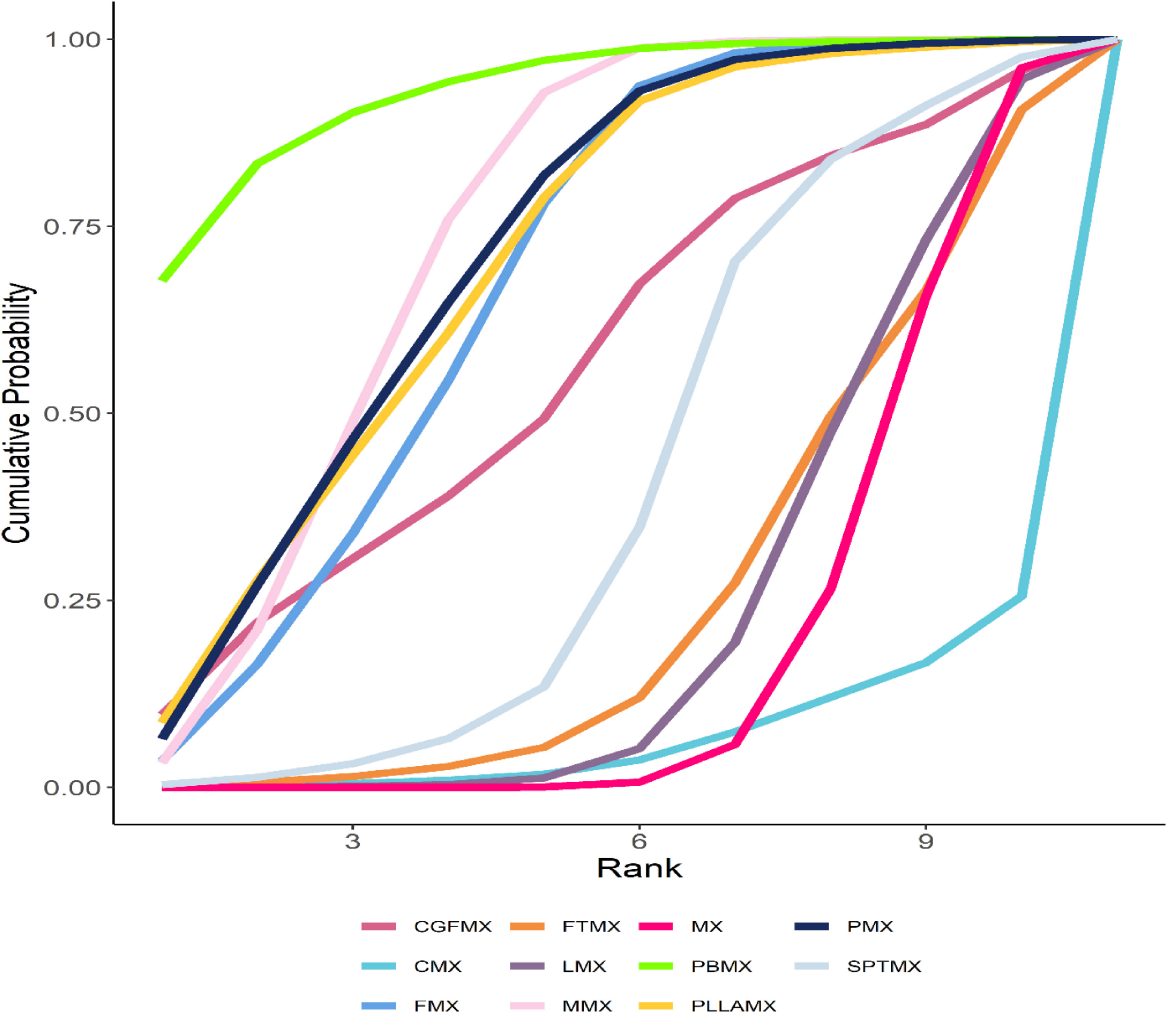
Optimized SUCRA folding plot for mixed groups. The area under the fold line represents the size of the sucra value, the better the efficacy, the larger the area.

Among the 20 study groups, the PBMX group demonstrated the highest overall efficacy, with a SUCRA value of 93.06%. In comparison to the group receiving minoxidil alone, the PBMX group exhibited a mean increase in hair density of 35.12 hairs/cm^2^. The group treated with microneedling combined with minoxidil showed an increase of 22.64 hairs/cm^2^ (SUCRA = 74.06%), while the PMX group had an increase of 22.14 hairs/cm^2^ (SUCRA = 71.53%). However, the study did not reveal any statistically significant differences in efficacy between the PBMX, MMX, and PMX groups. Most combination therapies demonstrated greater efficacy than the minoxidil alone group, with the exception of the cetirizine and minoxidil combination, which had a SUCRA value of 6.90%, as detailed in Supplementary Tables 4, 7. We refined the original SUCRA chart and represented it as a line graph, where a larger area under the curve corresponds to a higher SUCRA value and greater efficacy, as illustrated in Figure 4.

### Subgroup analysis

Given that the majority of research on androgenetic alopecia has been conducted separately for male and female subjects, subgroup analyses were performed based on the gender of the study participants. The network plots for these subgroups are presented in Supplementary Figures 1, 2.

In the male cohort, a total of five combined interventions were evaluated. The most efficacious treatment was the combination of finasteride and minoxidil, with a SUCRA value of 80.21%. This treatment resulted in an increase in hair density of 29.68 hairs/cm^2^ after 24 weeks, compared to the reference group. The second most effective treatment was the PMX group, with a SUCRA value of 73.00%, which achieved an increase in hair density of 27.18 hairs/cm^2^. Among male patients with androgenetic alopecia, all combination therapies demonstrated enhanced efficacy; however, only the FMX group exhibited a statistically significant difference in efficacy when compared to minoxidil alone, with the evidence being of moderate quality. The SUCRA ranking and corresponding league tables are provided in Supplementary Tables 5, 8, respectively, and the optimized fold plot is illustrated in Supplementary Figure 3.

Among the seven combination therapies evaluated in the female subgroup, the most effective treatments were microneedle combined with minoxidil (SUCRA = 87.20%) and silk thread combined with minoxidil (SUCRA = 84.51%). These combinations resulted in an increase in hair density of 22.02 hairs/cm^2^ and 21.63 hairs/cm^2^, respectively, after 24 weeks compared to minoxidil alone, with a statistically significant difference observed in the microneedle-minoxidil group, supported by moderate quality of evidence. The efficacy of spironolactone combined with minoxidil (SUCRA = 56.63%) and platelet-rich plasma combined with minoxidil (SUCRA = 53.88%) was comparable and also demonstrated superiority over minoxidil alone (SUCRA = 36.00%). Conversely, the combination of low-level light therapy and cetirizine with minoxidil exhibited reduced efficacy compared to their use as monotherapies. The quality of evidence within the female subgroups varied from very low to low, with only two comparisons achieving a moderate quality of evidence, as illustrated in Supplementary Figure 4 and Supplementary Tables 6, 9.

## Discussion

Hair loss can result from various causes, with androgenetic alopecia (AGA) being the most prevalent. AGA significantly affects patients’ quality of life and may lead to severe psychological disorders (30). While Minoxidil is an FDA-approved standard treatment for AGA, its efficacy as a monotherapy often falls short of patient expectations. Research indicates that combination therapies are generally more effective than monotherapy for treating AGA (31). In our study, the majority of combination therapies demonstrated superior efficacy compared to Minoxidil alone, with PBMX emerging as the most effective treatment (SUCRA = 93.00%). Previous network meta-analyses have also shown similar results when comparing Minoxidil combined with microneedling or platelet-rich plasma (32). Our study, however, included a more extensive and comprehensive range of combination therapies, all of which were evaluated through RCTs, thereby enhancing the credibility of our findings. Although PBMX exhibited the highest efficacy, no significant difference in relative effectiveness was observed when compared to the combination of microneedling and Minoxidil (SUCRA = 74.10%) and platelet-rich plasma combined with minoxidil (SUCRA = 71.68%).

The pathogenesis of androgenetic alopecia is primarily attributed to an exaggerated response to androgens, particularly dihydrotestosterone (DHT) and 5-alpha-reductase type II (3, 33). Given that treatment protocols differ between men and women, and most clinical trials are conducted separately for each gender, our study accounted for these differences by analyzing subgroups based on gender.

In men with male pattern baldness, finasteride, a 5-alpha reductase inhibitor, has been shown to effectively reduce dihydrotestosterone levels, with prior research indicating that oral finasteride is the most efficacious treatment (34). Our analysis revealed that for men, the combination of finasteride and minoxidil was the most effective treatment modality, demonstrating a significant difference compared to minoxidil alone. However, the combination of finasteride and minoxidil did not show a significant difference when compared to other combination therapies. It is important to note that our findings were derived from indirect comparisons, and the quality of the evidence was predominantly very low to low. Therefore, further direct comparison studies are necessary to validate our results.

In the female subgroup, the efficacy ranking indicated that the most effective treatment modality was the combination of microneedling and minoxidil. Consistent with findings in the male subgroup, the relative effectiveness of microneedling combined with minoxidil did not significantly differ from other combination therapies. Our NMA revealed that two regimens were less effective than minoxidil alone; however, these differences were not statistically significant. Given that our study exclusively included RCTs, resulting in small sample sizes for each therapy, further RCTs are necessary to substantiate these conclusions.

### Strengths and limitations

The relative efficacy of minoxidil combined with microneedling or platelet-rich plasma has been examined in only one prior study. For our analysis, we intentionally included only randomized controlled trials to enhance the reliability of our findings and consistently selected a 24-weeks follow-up period to reduce selection bias associated with varying outcome assessment time points. Our study not only assessed overall efficacy but also conducted subgroup analyses to investigate gender-specific effects in androgenic alopecia, thereby aiming to derive more comprehensive conclusions.

Nonetheless, our research has certain limitations. Firstly, the small sample size and the lack of direct comparisons among various combination therapies resulted in a low quality of evidence for relative comparisons. Therefore, larger sample sizes and more direct comparative studies are necessary to validate these findings in future research. Secondly, a significant portion of the existing literature does not explicitly detail the distribution of different severities of androgenic alopecia, which may have influenced our results. Thirdly, while dutasteride and oral minoxidil have demonstrated promising efficacy in treating AGA, our NMA lacks studies on these two medications. Future research should include these drugs to facilitate a more comprehensive comparative assessment of combination therapy efficacy. Lastly, the insufficient characterization of AGA severity in existing studies precludes further exploration of the impact of disease severity on treatment efficacy, which may affect the reliability of our results.

## Conclusion

This study conducted an analysis to ascertain the relative efficacy rankings of minoxidil-based combination therapies, thereby offering evidence-based guidance for dermatologists aiming to improve the management of androgenetic alopecia. Nonetheless, additional research, particularly direct comparative studies of various combination therapies, is necessary to validate these findings.

## Data Availability

The raw data supporting the conclusions of this article will be made available by the authors, without undue reservation.
