# Supplementary material for: Relative efficacy of minoxidil in combination with other treatments for androgenic alopecia: a network meta-analysis based on randomized controlled trials
Source: Front Med (Lausanne). 2025 Sep 17;12:1638496. doi: 10.3389/fmed.2025.1638496 (PMC12483851; doi:10.3389/fmed.2025.1638496)
Supplement: Supplementary file 1 [file Table_1.docx]

| Table S1. Assessment of Quality of Evidence for Mixed Groups |
| --- |
| \| Comparison \| Number of studies \| Confidence rating \| \| --- \| --- \| --- \| \| LMX: MX \| 4 \| Moderate \| \| PLLAMX: MX \| 1 \| Very Low \| \| PMX: MX \| 2 \| Moderate \| \| PBMX: MX \| 1 \| Moderate \| \| MMX: MX \| 4 \| Moderate \| \| FMX: MX \| 3 \| High \| \| FTMX: MX \| 1 \| Very Low \| \| CMX: MX \| 1 \| Very Low \| \| CGFMX: MX \| 1 \| Moderate \| \| SPTMX: MX \| 1 \| Moderate \| \| LMX: PLLAMX \| 0 \| Very Low \| \| LMX: PMX \| 0 \| Low \| \| LMX: PBMX \| 0 \| Very Low \| \| LMX: MMX \| 0 \| Low \| \| LMX: FMX \| 0 \| Low \| \| LMX: FTMX \| 0 \| Very Low \| \| LMX: CMX \| 0 \| Very Low \| \| LMX: CGFMX \| 0 \| Low \| \| LMX: SPTMX \| 0 \| Low \| \| PLLAMX: PMX \| 0 \| Very Low \| \| PLLAMX: PBMX \| 0 \| Very Low \| \| PLLAMX: MMX \| 0 \| Very Low \| \| PLLAMX: FMX \| 0 \| Low \| \| PLLAMX: FTMX \| 0 \| Very Low \| \| PLLAMX: CMX \| 0 \| Very Low \| \| PLLAMX: CGFMX \| 0 \| Very Low \| \| PLLAMX: SPTMX \| 0 \| Very Low \| \| PMX: PBMX \| 0 \| Low \| \| PMX: MMX \| 0 \| Low \| \| PMX: FMX \| 0 \| Moderate \| \| PMX: FTMX \| 0 \| Very Low \| \| PMX: CMX \| 0 \| Very Low \| \| PMX: CGFMX \| 0 \| Low \| \| PMX: SPTMX \| 0 \| Low \| \| PBMX: MMX \| 0 \| Low \| \| PBMX: FMX \| 0 \| Moderate \| \| PBMX: FTMX \| 0 \| Very Low \| \| PBMX: CMX \| 0 \| Very Low \| \| PBMX: CGFMX \| 0 \| Low \| \| PBMX: SPTMX \| 0 \| Low \| \| MMX: FMX \| 0 \| Moderate \| \| MMX: FTMX \| 0 \| Very Low \| \| MMX: CMX \| 0 \| Very Low \| \| MMX: CGFMX \| 0 \| Low \| \| MMX: SPTMX \| 0 \| Low \| \| FMX: FTMX \| 0 \| Very Low \| \| FMX: CMX \| 0 \| Very Low \| \| FMX: CGFMX \| 0 \| Low \| \| FMX: SPTMX \| 0 \| Moderate \| \| FTMX: CMX \| 0 \| Very Low \| \| FTMX: CGFMX \| 0 \| Very Low \| \| FTMX: SPTMX \| 0 \| Very Low \| \| CMX: CGFMX \| 0 \| Very Low \| \| CMX: SPTMX \| 0 \| Very Low \| \| CGFMX: SPTMX \| 0 \| Low \| |

| Table S2. Assessment of Quality of Evidence for Male Subgroups |
| --- |
| \| Comparison \| Number of studies \| Confidence rating \| \| --- \| --- \| --- \| \| LMX: MX \| 2 \| Low \| \| PMX: MX \| 1 \| Moderate \| \| MMX: MX \| 2 \| Moderate \| \| FMX: MX \| 2 \| Moderate \| \| CGFMX: MX \| 1 \| Moderate \| \| LMX: PMX \| 0 \| Very Low \| \| LMX: MMX \| 0 \| Very Low \| \| LMX: FMX \| 0 \| Very Low \| \| LMX: CGFMX \| 0 \| Very Low \| \| PMX: MMX \| 0 \| Low \| \| PMX: FMX \| 0 \| Low \| \| PMX: CGFMX \| 0 \| Low \| \| MMX: FMX \| 0 \| Low \| \| MMX: CGFMX \| 0 \| Low \| \| FMX: CGFMX \| 0 \| Low \| |

| Table S3. Assessment of Quality of Evidence for Female Subgroups |
| --- |
| \| Comparison \| Number of studies \| Confidence rating \| \| --- \| --- \| --- \| \| LMX: MX \| 1 \| Low \| \| PLLAMX: MX \| 1 \| Very Low \| \| FMX: MX \| 1 \| Low \| \| MMX: MX \| 2 \| Moderate \| \| CMX: MX \| 1 \| Very Low \| \| PMX: MX \| 1 \| Very Low \| \| SPTMX: MX \| 1 \| Moderate \| \| LMX: PLLAMX \| 0 \| Very Low \| \| LMX: FMX \| 0 \| Very Low \| \| LMX: MMX \| 0 \| Low \| \| LMX: CMX \| 0 \| Very Low \| \| LMX: PMX \| 0 \| Very Low \| \| LMX: SPTMX \| 0 \| Low \| \| PLLAMX: FMX \| 0 \| Very Low \| \| PLLAMX: MMX \| 0 \| Very Low \| \| PLLAMX: CMX \| 0 \| Very Low \| \| PLLAMX: PMX \| 0 \| Very Low \| \| PLLAMX: SPTMX \| 0 \| Very Low \| \| FMX: MMX \| 0 \| Low \| \| FMX: CMX \| 0 \| Very Low \| \| FMX: PMX \| 0 \| Very Low \| \| FMX: SPTMX \| 0 \| Low \| \| MMX: CMX \| 0 \| Very Low \| \| MMX: PMX \| 0 \| Very Low \| \| MMX: SPTMX \| 0 \| Low \| \| CMX: PMX \| 0 \| Very Low \| \| CMX: SPTMX \| 0 \| Very Low \| \| PMX: SPTMX \| 0 \| Very Low \| |

| Figure S1. Network plot for the male subgroup |
| --- |
| 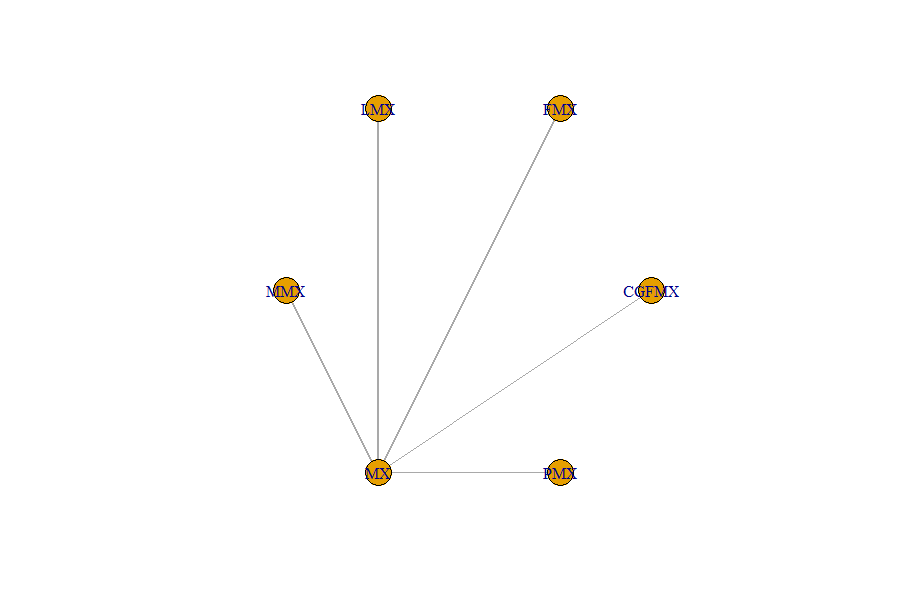 |
| One node corresponds to a given intervention; An edge is represented by a line between the two nodes, which corresponds to a direct comparison of the two interventions in a head-on trial. The thickness of an edge corresponds to the number of direct comparisons between the respective nodes. |

| Figure S2. Network plot for the female subgroup |
| --- |
| 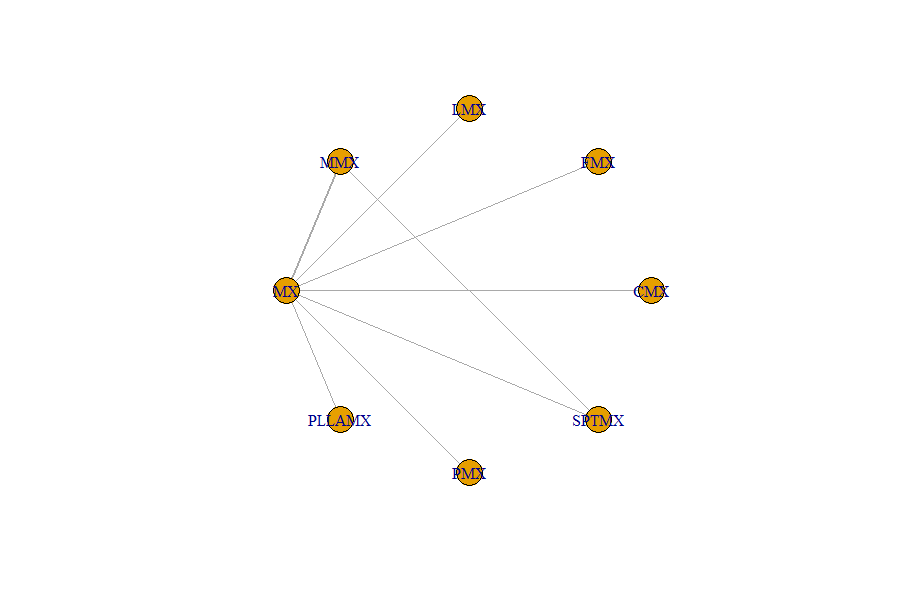 |
| One node corresponds to a given intervention; An edge is represented by a line between the two nodes, which corresponds to a direct comparison of the two interventions in a head-on trial. The thickness of an edge corresponds to the number of direct comparisons between the respective nodes. |

| Table S4. League tables are sorted by SUCRA value for mixed groups. |
| --- |
| \|  \| CMX \| MX \| LMX \| FTMX \| SPTMX \| CGFMX \| FMX \| PLLAMX \| PMX \| MMX \| PBMX \| \| --- \| --- \| --- \| --- \| --- \| --- \| --- \| --- \| --- \| --- \| --- \| --- \| \| CMX \| CMX \| 13.37 (-13.44, 40.59) \| 14.55 (-14.28, 43.29) \| 14.71 (-17.22, 47.5) \| 21.12 (-10.39, 53.22) \| 29.01 (-11.62, 69.76) \| 33.65 (3.61, 64.24) \| 35.04 (1.98, 68.59) \| 35.35 (3.1, 65.85) \| 35.95 (7.69, 64.77) \| 48.5 (14.45, 83.11) \| \| MX \| -13.37 (-40.59, 13.44) \| MX \| 1.21 (-9.51, 11.01) \| 1.37 (-16.81, 19.8) \| 7.79 (-9.26, 24.9) \| 15.63 (-14.28, 45.65) \| 20.34 (6.39, 34.16) \| 21.73 (2.02, 41.4) \| 22.14 (4.3, 36.63) \| 22.64 (12.99, 32.15) \| 35.12 (13.87, 56.41) \| \| LMX \| -14.55 (-43.29, 14.28) \| -1.21 (-11.01, 9.51) \| LMX \| 0.14 (-20.17, 21.86) \| 6.54 (-12.79, 27.02) \| 14.44 (-16.81, 46.39) \| 19.16 (2.14, 36.73) \| 20.5 (-1.26, 43.24) \| 20.96 (0.64, 38.61) \| 21.43 (7.69, 35.87) \| 33.95 (10.75, 57.99) \| \| FTMX \| -14.71 (-47.5, 17.22) \| -1.37 (-19.8, 16.81) \| -0.14 (-21.86, 20.17) \| FTMX \| 6.4 (-18.61, 31.57) \| 14.17 (-20.26, 49.09) \| 19.04 (-3.97, 41.7) \| 20.28 (-6.63, 47.22) \| 20.9 (-5.58, 43.17) \| 21.28 (0.42, 41.83) \| 33.75 (5.81, 61.62) \| \| SPTMX \| -21.12 (-53.22, 10.39) \| -7.79 (-24.9, 9.26) \| -6.54 (-27.02, 12.79) \| -6.4 (-31.57, 18.61) \| SPTMX \| 7.81 (-26.19, 42.18) \| 12.59 (-9.52, 34.42) \| 13.94 (-12.32, 39.95) \| 14.45 (-11.07, 35.9) \| 14.86 (-2.38, 31.89) \| 27.33 (0.22, 54.59) \| \| CGFMX \| -29.01 (-69.76, 11.62) \| -15.63 (-45.65, 14.28) \| -14.44 (-46.39, 16.81) \| -14.17 (-49.09, 20.26) \| -7.81 (-42.18, 26.19) \| CGFMX \| 4.65 (-28.28, 37.53) \| 6.16 (-29.61, 41.66) \| 6.3 (-28.6, 39.14) \| 6.99 (-24.52, 38.27) \| 19.45 (-17.11, 56.18) \| \| FMX \| -33.65 (-64.24, -3.61) \| -20.34 (-34.16, -6.39) \| -19.16 (-36.73, -2.14) \| -19.04 (-41.7, 3.97) \| -12.59 (-34.42, 9.52) \| -4.65 (-37.53, 28.28) \| FMX \| 1.38 (-22.59, 25.35) \| 1.8 (-21.28, 21.51) \| 2.27 (-14.59, 19.1) \| 14.79 (-10.69, 40.34) \| \| PLLAMX \| -35.04 (-68.59, -1.98) \| -21.73 (-41.4, -2.02) \| -20.5 (-43.24, 1.26) \| -20.28 (-47.22, 6.63) \| -13.94 (-39.95, 12.32) \| -6.16 (-41.66, 29.61) \| -1.38 (-25.35, 22.59) \| PLLAMX \| 0.46 (-26.84, 24.09) \| 0.93 (-21.08, 22.72) \| 13.42 (-15.67, 42.42) \| \| PMX \| -35.35 (-65.85, -3.1) \| -22.14 (-36.63, -4.3) \| -20.96 (-38.61, -0.64) \| -20.9 (-43.17, 5.58) \| -14.45 (-35.9, 11.07) \| -6.3 (-39.14, 28.6) \| -1.8 (-21.51, 21.28) \| -0.46 (-24.09, 26.84) \| PMX \| 0.44 (-16.63, 20.92) \| 12.98 (-12.13, 41.51) \| \| MMX \| -35.95 (-64.77, -7.69) \| -22.64 (-32.15, -12.99) \| -21.43 (-35.87, -7.69) \| -21.28 (-41.83, -0.42) \| -14.86 (-31.89, 2.38) \| -6.99 (-38.27, 24.52) \| -2.27 (-19.1, 14.59) \| -0.93 (-22.72, 21.08) \| -0.44 (-20.92, 16.63) \| MMX \| 12.47 (-10.66, 35.86) \| \| PBMX \| -48.5 (-83.11, -14.45) \| -35.12 (-56.41, -13.87) \| -33.95 (-57.99, -10.75) \| -33.75 (-61.62, -5.81) \| -27.33 (-54.59, -0.22) \| -19.45 (-56.18, 17.11) \| -14.79 (-40.34, 10.69) \| -13.42 (-42.42, 15.67) \| -12.98 (-41.51, 12.13) \| -12.47 (-35.86, 10.66) \| PBMX \| |
| This figure presents all pairwise comparisons in the network for change in hair/cm2 after 24 weeks of therapy. Each point estimate represents the mean difference, and the values in parentheses are the corresponding 95% credible interval. Yellow-colored cells represent significant comparisons; each cell compares the column against the row. |

| Table S5. League tables are sorted by SUCRA value for male subgroups. |
| --- |
| \|  \| MX \| LMX \| CGFMX \| MMX \| PMX \| FMX \| \| --- \| --- \| --- \| --- \| --- \| --- \| --- \| \| MX \| MX \| 5.43 (-20.34, 28.19) \| 15.77 (-23.53, 55.25) \| 23.05 (-0.98, 45.79) \| 27.18 (-6.5, 61.08) \| 29.68 (5.74, 59.48) \| \| LMX \| -5.43 (-28.19, 20.34) \| LMX \| 10.56 (-34.34, 57.84) \| 17.47 (-15.37, 52.38) \| 21.7 (-18.12, 64.71) \| 24.22 (-7.45, 65.22) \| \| CGFMX \| -15.77 (-55.25, 23.53) \| -10.56 (-57.84, 34.34) \| CGFMX \| 7.07 (-38.71, 52.1) \| 11.44 (-40.06, 62.91) \| 14.17(-31.03, 63.77) \| \| MMX \| -23.05 (-45.79, 0.98) \| -17.47 (-52.38, 15.37) \| -7.07 (-52.1, 38.71) \| MMX \| 4.22 (-36.18, 45.94) \| 6.63 (-25.42, 46.01) \| \| PMX \| -27.18 (-61.08, 6.5) \| -21.7 (-64.71, 18.12) \| -11.44 (-62.91, 40.06) \| -4.22 (-45.94, 36.18) \| PMX \| 2.42 (-37.41, 48.86) \| \| FMX \| -29.68 (-59.48, -5.74) \| -24.22 (-65.22, 7.45) \| -14.17 (-63.77, 31.03) \| -6.63 (-46.01, 25.42) \| -2.42 (-48.86, 37.41) \| FMX \| |
| This figure presents all pairwise comparisons in the network for change in hair/cm2 after 24 weeks of therapy. Each point estimate represents the mean difference, and the values in parentheses are the corresponding 95% credible interval. Yellow-colored cells represent significant comparisons; each cell compares the column against the row. |

| Table S6. League tables are sorted by SUCRA value for female subgroups. |
| --- |
| \|  \| CMX \| LMX \| MX \| FMX \| PMX \| SPTMX \| PLLAMX \| MMX \| \| --- \| --- \| --- \| --- \| --- \| --- \| --- \| --- \| --- \| \| CMX \| CMX \| 6.45 (-35.05, 48.04) \| 13.98 (-17.26, 45.13) \| 16.97 (-25.38, 58.56) \| 21.4 (-23.88, 66.44) \| 21.48 (-17.78, 60.71) \| 35.63 (-5.04, 75.99) \| 36.07 (0.25, 72.26) \| \| LMX \| -6.45 (-48.04, 35.05) \| LMX \| 7.45 (-20.02, 34.87) \| 10.44 (-29.21, 49.69) \| 14.77 (-27.54, 57.17) \| 14.91 (-21.13, 51.81) \| 29.1 (-8.8, 67.05) \| 29.47 (-3.24, 62.86) \| \| MX \| -13.98 (-45.13, 17.26) \| -7.45 (-34.87, 20.02) \| MX \| 3.01 (-25.44, 31.15) \| 7.38 (-25.15, 39.64) \| 7.44 (-16.45, 32.07) \| 21.63 (-4.6, 47.95) \| 22.02 (3.92, 40.95) \| \| FMX \| -16.97 (-58.56, 25.38) \| -10.44 (-49.69, 29.21) \| -3.01 (-31.15, 25.44) \| FMX \| 4.43 (-38.39, 47.5) \| 4.46 (-32.23, 42.17) \| 18.61 (-19.6, 57.45) \| 19.04 (-14.21, 53.24) \| \| PMX \| -21.4 (-66.44, 23.88) \| -14.77 (-57.17, 27.54) \| -7.38 (-39.64, 25.15) \| -4.43 (-47.5, 38.39) \| PMX \| 0.11 (-39.66, 40.29) \| 14.24 (-26.99, 55.9) \| 14.71 (-22.17, 52.25) \| \| SPTMX \| -21.48 (-60.71, 17.78) \| -14.91 (-51.81, 21.13) \| -7.44 (-32.07, 16.45) \| -4.46 (-42.17, 32.23) \| -0.11 (-40.29, 39.66) \| SPTMX \| 14.19 (-21.79, 49.58) \| 14.54 (-9.54, 39.2) \| \| PLLAMX \| -35.63 (-75.99, 5.04) \| -29.1 (-67.05, 8.8) \| -21.63 (-47.95, 4.6) \| -18.61 (-57.45, 19.6) \| -14.24 (-55.9, 26.99) \| -14.19 (-49.58, 21.79) \| PLLAMX \| 0.35 (-31.23, 32.81) \| \| MMX \| -36.07 (-72.26, -0.25) \| -29.47 (-62.86, 3.24) \| -22.02 (-40.95, -3.92) \| -19.04 (-53.24, 14.21) \| -14.71 (-52.25, 22.17) \| -14.54 (-39.2, 9.54) \| -0.35 (-32.81, 31.23) \| MMX \| |
| This figure presents all pairwise comparisons in the network for change in hair/cm2 after 24 weeks of therapy. Each point estimate represents the mean difference, and the values in parentheses are the corresponding 95% credible interval. Yellow-colored cells represent significant comparisons; each cell compares the column against the row. |

| Table S7. SUCRA values for mixed groups |
| --- |
| \| REGMEN \| SUCRA（%） \| \| --- \| --- \| \| PBMX \| 93.06 \| \| MMX \| 74.06 \| \| PMX \| 71.53 \| \| PLLAMX \| 70.52 \| \| FMX \| 67.76 \| \| CGFMX \| 56.56 \| \| SPTMX \| 40.28 \| \| FTMX \| 25.66 \| \| LMX \| 24.17 \| \| MX \| 19.50 \| \| CMX \| 6.90 \| |

| Table S8. SUCRA values for male subgroups |
| --- |
| \| REGMEN \| SUCRA（%） \| \| --- \| --- \| \| FMX \| 80.21 \| \| PMX \| 73.00 \| \| MMX \| 63.28 \| \| CGFMX \| 48.01 \| \| LMX \| 24.92 \| \| MX \| 10.56 \| |

| Table S9. SUCRA values for female subgroups |
| --- |
| \| REGMEN \| SUCRA（%） \| \| --- \| --- \| \| MMX \| 87.20 \| \| PLLAMX \| 84.51 \| \| SPTMX \| 56.63 \| \| PMX \| 53.88 \| \| FMX \| 44.59 \| \| MX \| 36.00 \| \| LMX \| 22.23 \| \| CMX \| 14.96 \| |

| Figure S3. Optimized SUCRA Folding Plot For Male Subgroups |
| --- |
| 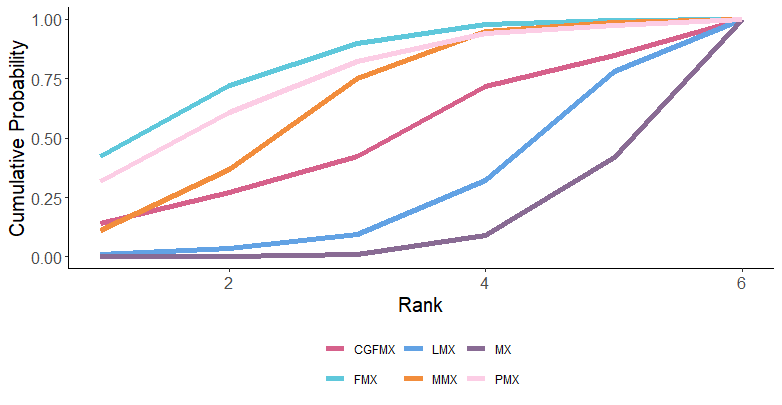 |
| The area under the fold line represents the size of the SUCRA value, the better the efficacy, the larger the area |

| Figure S4. Optimized SUCRA Folding Plot For Female Subgroups |
| --- |
| 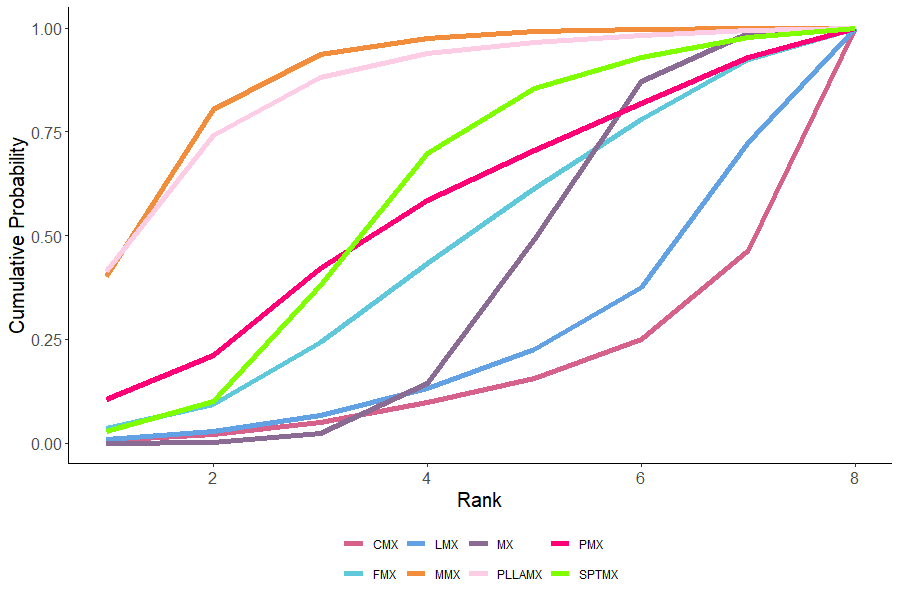 |
| The area under the fold line represents the size of the SUCRA value, the better the efficacy, the larger the area |
